# SDG indicator 3.b.3 – an analysis of its robustness and challenges for measuring access to medicines for children

**DOI:** 10.1186/s12913-023-09554-w

**Published:** 2023-06-03

**Authors:** I. R. Joosse, V. J. Wirtz, A. T. van Mourik, B. A. Wagner, A. K. Mantel-Teeuwisse, F. Suleman, H. A. van den Ham

**Affiliations:** 1 Utrecht WHO Collaborating Centre for Pharmaceutical Policy and Regulation, Division of Pharmacoepidemiology and Clinical Pharmacology, Utrecht Institute for Pharmaceutical Sciences (UIPS), Utrecht University, Universiteitsweg 99, 3584 CG Utrecht, the Netherlands; 2 WHO Collaborating Centre in Pharmaceutical Policy, Department of Global Health, Boston University School of Public Health, Boston, USA; 3 WHO Collaborating Centre for Pharmaceutical Policy and Evidence Based Practice, School of Health Sciences, University of KwaZulu-Natal, Durban, South Africa

**Keywords:** Access to medicines, Child medicines, Child health, Sustainable development goals, Indicator, Affordability, Availability

## Abstract

**Background:**

Sustainable Development Goal (SDG) indicator 3.b.3 monitors progress in medicines’ accessibility for adults and has significant limitations when applying to medicines for children. An adapted indicator methodology was developed to fill this gap, but no proof of its robustness exists. We provide this evidence through sensitivity analyses.

**Methods:**

Data on availability and prices of child medicines from ten historical datasets were combined to create datasets for analysis: Dataset 1 (medicines selected at random) and Dataset 2 (preference given to available medicines, to better capture affordability of medicines). A base case scenario and univariate sensitivity analyses were performed to test critical components of the methodology, including the new variable of number of units needed for treatment (NUNT), disease burden (DB) weighting, and the National Poverty Line (NPL) limits. Additional analyses were run on a continuously smaller basket of medicines to explore the minimum number of medicines required. Mean facility scores for access were calculated and compared.

**Results:**

The mean facility score for Dataset 1 and Dataset 2 within the base case scenario was 35.5% (range 8.0–58.8%) and 76.3% (range 57.2–90.6%). Different NUNT scenarios led to limited variations in mean facility scores of + 0.1% and -0.2%, or differences of + 4.4% and -2.1% at the more critical NPL of $5.50 (Dataset 1). For Dataset 2, variations to the NUNT generated differences of + 0.0% and -0.6%, at an NPL of $5.50 the differences were + 5.0 and -2.0%. Different approaches for weighting for DB induced considerable fluctuations of 9.0% and 11.2% respectively. Stable outcomes with less than 5% change in mean facility score were observed for a medicine basket down to 12 medicines. For smaller baskets, scores increased more rapidly with a widening range.

**Conclusion:**

This study has confirmed that the proposed adaptations to make SDG indicator 3.b.3 appropriate for children are robust, indicating that they could be an important addition to the official Global Indicator Framework. At least 12 child-appropriate medicines should be surveyed to obtain meaningful outcomes. General concerns that remain about the weighting of medicines for DB and the NPL should be considered at the 2025 planned review of this framework.

**Supplementary Information:**

The online version contains supplementary material available at 10.1186/s12913-023-09554-w.

## Introduction

Despite the considerable progress in child health that has been achieved in recent decades, high child morbidity and mortality rates remain an urgent challenge globally [1]. Recent data suggests that more than 50 countries worldwide will fail to meet the targets set under Sustainable Development Goal (SDG) 3.2 to end preventable deaths of children by 2030 [2]. Limited availability of affordable essential medicines for children contributes to child mortality. To improve this outcome, access to these medicines has been recognized as an important priority, outlined in SDG targets 3.8 and 3.b [3]. Measurement and monitoring of access to medicines is an integral part of this, and will aid national and international policy-makers in directing their efforts and formulating effective policies.

**Fig. 1:**
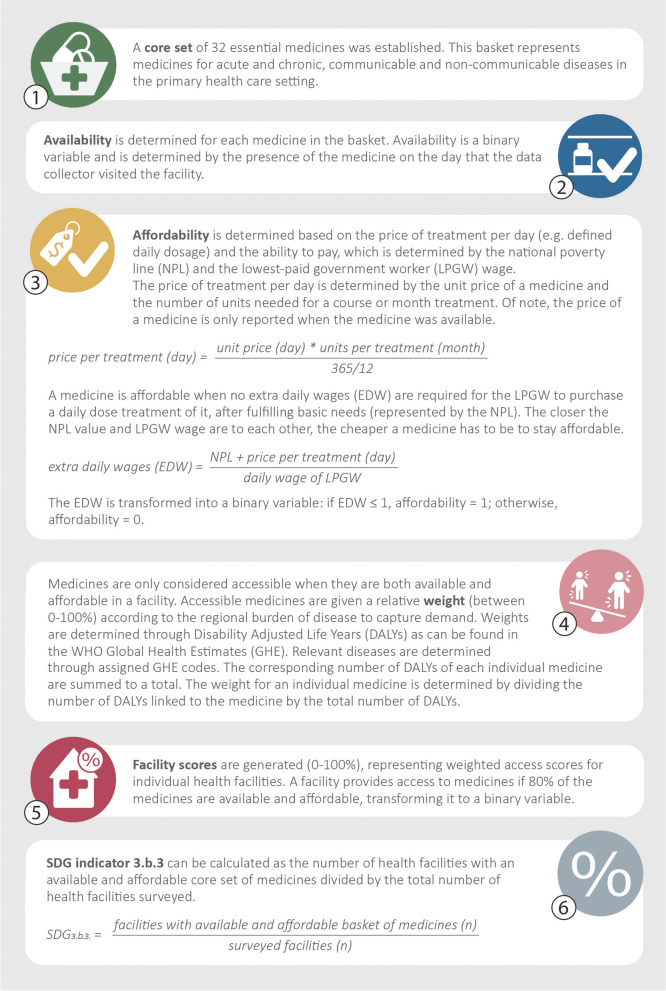
Critical steps in calculating access to medicines with SDG indicator 3.b.3. Adapted from United Nations [4]

The United Nations (UN) under the leadership of the World Health Organization (WHO) has developed SDG indicator 3.b.3 to track progress on access to medicines (Fig. 1) [4]. The novelty and significance of this indicator versus established methods for measuring access to medicines (e.g. the WHO/Health Action International (HAI) methodology) lies in the combined analysis of two crucial dimensions of access: availability and affordability. Indicator 3.b.3 has been part of the official Global Indicator Framework since 2017 and was re-classified in 2018 as a Tier II indicator, which means that the indicator is conceptually clear and has an established methodology, but data are not regularly produced by countries [5, 6].

Although access to medicines for children has recently been reported on in the context of the SDG 3 targets, the methodology as developed for indicator 3.b.3 was not employed [7]. This may be explained by the unfitness of this indicator for measuring access to child medicines, since it fails to address the unique requirements of children. Specifically, the indicator chiefly targets typical adult diseases such as type II diabetes and cardiovascular diseases, it fails to consider age-appropriate formulations, and the methodology depends on defined daily dosages (DDDs) to express affordability, which applies to adults only [8]. To address this gap and enable the measuring of access to child medicines, a conceptual methodology was developed based on the principles embedded in the existing SDG Indicator 3.b.3 [9]. Although proof-of concept for this adapted methodology was provided by applying the method to three historical datasets, the robustness of the adapted indicator could not be established in this pilot due to a lack of data on pediatric medicines in these datasets.

Before the child methodology can be applied on a larger scale, several validation steps must be undertaken to ensure the robustness of the methodology. This is of particular importance for the NUNT (i.e. Number of Units Needed for Treatment), a novel parameter that was introduced in the adapted methodology to substitute DDDs in the calculation of affordability of medicines and incorporates the dosages required by children of different ages. In addition to this, sensitivity analyses should reveal how many child medicines need to be surveyed for a reliable measure of access.

Besides these validation steps, questions on the general framework of the indicator that were raised in previous research also call for further study [9]. These include concerns about the weighting for regional disease burden (DB) parameter (see Fig. 1). This step was inserted when developing indicator 3.b.3 to increase the specificity of a global basket of medicines to a national context. However, there are concerns that the current weighting approach has introduced disproportionality due to 1) higher proportional contribution for indications for which there are multiple medicines in the basket and 2) antibacterial medicines that are weighted for indications for which they are not used. Other questions raised pertained to expressing affordability as a function of the National Poverty Line (NPL) in addition to the Lowest Paid unskilled Government Worker (LPGW) wage.

The aim of the present study is to determine the robustness of the adapted SDG indicator 3.b.3 methodology for children and to address remaining concerns through sensitivity analyses. This will not only help validate the adapted methodology for children but will also contribute to our understanding of the main SDG indicator 3.b.3.

## Methods

To make indicator 3.b.3 appropriate for children, adaptations to the methodology presented in Fig. 1 encompassed 1) the selection of two new baskets of medicines for prevalent child diseases – including age-appropriate strengths and formulations for young children aged 1 month to 5 years and for school-aged children aged 5–12 years – and 2) the establishment of the NUNT [4]. A third adaptation tested in this proof-of-concept study pertained to the weighting for DB. Global Health Estimates (GHE) code 370 (for 'other infectious diseases') was used instead of code 20 (for ‘infectious and parasitic diseases’) for antibacterial medicines, because the latter code encompasses diseases such as hepatitis for which these medicines are not used. A detailed description of the adapted indicator methodology can be found in Annex 1, including the core set of medicines for children 1–59 months that should be used in the calculation of indicator 3.b.3 (Table S1).

### Data selection

The present study focused on children aged one month to five years. To secure sufficient availability and price data on eligible medicines for this age group for conducting meaningful sensitivity analyses, ten historical WHO/HAI datasets from eight countries (Bolivia (2008), Burundi (2013), China (2012), Haiti (2011), Kyrgyzstan (2010, 2015), Mongolia (2004), Sudan (2012, 2013), Tanzania (2012)) were combined into a single database [10]. Data on eligible medicines (i.e. those listed in Table S1) from the different datasets were pooled to constitute 25 hypothetical facilities. In the pooling process, facilities from different datasets were matched to each other on sector and level of care as closely as possible. The resulting 25 hypothetical facilities each contained data on a range of eligible child formulations within the same therapeutic class, and often included duplicates of formulations due to the pooling of data from multiple countries. All data were corrected for inflation using the Consumer Price Index (CPI) and purchasing power parity (PPP) [11, 12].

From the resultant database, two distinct datasets were extracted. For Dataset 1, one medicine formulation per therapeutic class was extracted at random if medicines were interchangeable, irrespective of whether availability or price information was complete. In case of duplicates, data from one country was selected at random. In the extraction process for a second dataset (Dataset 2), a purposeful sampling strategy giving preference to medicines with data on price (i.e. medicines that had been available) was used. This second approach was chosen, because we hypothesized that it enabled more thorough analysis of the affordability dimension of the methodology. Each of the two datasets was composed of data on 19 medicines across 25 health facilities.

### Additional data sources

Calculation of the SDG 3.b.3 indicator for children also requires data on the NUNT, NPLs, the LPGW wage and DB. The NUNT was predetermined for all medicine formulations in the basket and is based on the recommended dose and duration of treatment for an average child within the age group (Annex 2). To investigate the robustness of this single parameter as a way to represent an entire age group, a minimum and maximum NUNT were also established.

NPL values for each of the eight countries that the datasets originated from proved difficult to obtain as these were not readily available in the public domain (and may not exist for some countries), so international reference poverty lines were used instead. As the data originated from countries with different income levels, three international reference values of $1.90/day (for low-income countries), $3.20/day (for lower-middle income countries) and $5.50/day (for upper-middle income countries) were used to avoid misrepresentation [13]. We calculated a single (average) value for the LPGW wage, based on local LPGW wages as reported in the ten original datasets. These were corrected for CPI and PPP and averaged to $5.94/day (range $1.72–9.60) [11, 12].

Data on DB were extracted from the GHEs according to the codes as indicated in the predetermined basket of medicines (see Table S1 in Annex 1) [14].

### Sensitivity analyses

**Fig. 2:**
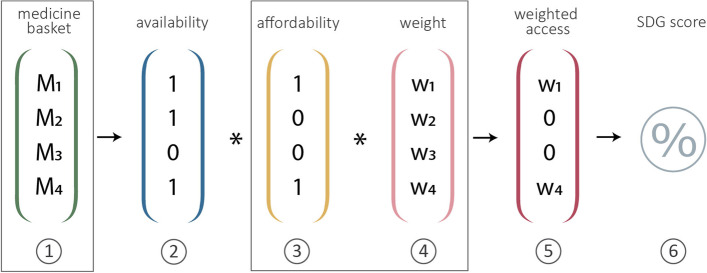
Matrix of weighted access to medicines in (adapted) SDG indicator 3.b.3. The weighted access equals the facility score. M_1-4_ = medicine 1–4, w_1-4_ = weight 1–4

To evaluate the robustness of the indicator methodology, we ran several scenarios with different input parameters to investigate the degree of variation in the outcomes. These sensitivity analyses targeted steps 1, 3 and 4 of the calculations as outlined in Fig. 2. It is through these respective steps that methodological choices could affect the outcomes, whereas steps 2, 5 and 6 are solely determined by the pattern of the underlying data. An overview of the various scenarios that were run is provided in Table 1.

**Table 1:** Overview of parameters and variations across scenarios

				**Expected effect compared to base case**	
**Scenario**	**DB**	**NPL**	**NUNT**	**on affordability**	**on facility scores**
A (base case)	GHE code 370 for antibacterials; DB of medicines used for the same disease counted multiple times	$1.90	Standard	NA	NA
B	GHE code 370 for antibacterials; DB of medicines used for the same disease divided by number of medicines	$1.90	Standard	No effect	Variable^c^
C^a^	GHE code 20 for antibacterials; DB of medicines used for the same disease counted multiple times	$1.90	Standard	No effect	Variable^c^
D	GHE code 20 for antibacterials; DB of medicines used for the same disease divided by number of medicines	$1.90	Standard	No effect	Variable^c^
E^b^	No DB weighting applied	$1.90	Standard	No effect	Variable^c^
F	As scenario A	$3.20	Standard	Decrease	Decrease
G	As scenario A	$5.50	Standard	Decrease	Decrease
H	As scenario A	$1.90	Minimum	Increase	Increase
I	As scenario A	$1.90	Maximum	Decrease	Decrease
J	As scenario A	$5.50	Minimum	Decrease^d^	Decrease^d^
K	As scenario A	$5.50	Maximum	Decrease^e^	Decrease^e^

*DB* Disease Burden, *NA* Not available, *NPL* National Poverty Line, *NUNT* Number of Units Needed for Treatment, *GHE* Global Health Estimates

^a^Scenario C is equivalent to the weighing system used in the main SDG indicator 3.b.3 methodology

^b^No DB weighting translates to equal weights for all medicines

^c^Effect (increase/decrease) depends on patterns in underlying data, e.g. which medicines are accessible

^d^Expected to increase compared to scenario G

^e^Expected to decrease compared to scenario G

For the base case scenario (scenario A), standard NUNT values, an NPL of $1.90 and DB weights based on GHE code 370 (plus additional disease-specific codes, see Annex 1) for antibacterial medicines were used. Across scenarios B to D, different approaches for calculating relative DB weights were tested (step 4 in Fig. 2). Proportional weights assigned across scenarios A-E are provided in Table 2. The influence of variations to the NPL, the NUNT or both were explored in scenarios F-K (step 3 in Fig. 2).

**Table 2:** Proportional weights (%) assigned to medicines in different burden of disease scenarios

Medicine	Scenario	A (base case)	B	C	D	E
		code 370; DB multiplied	code 370; DB divided	code 20; DB multiplied	code 20; DB divided	No weighting
Oral rehydration salts		8.5	12.1	3.9	5.6	5.3
Zinc sulphate		8.5	12.1	3.9	5.6	5.3
Phenytoin		0.3	0.3	0.1	0.1	5.3
Valproic acid		0.3	0.3	0.1	0.1	5.3
Diazepam		0.3	0.3	0.1	0.1	5.3
Ferrous salt		0.8	1.1	0.4	0.5	5.3
Mebendazole		0.8	1.1	0.4	0.5	5.3
Artemether + lumefantrine		6.2	8.8	2.9	4.1	5.3
Vitamin A		1.8	5.3	0.9	2.5	5.3
Paracetamol		4.5	4.0	4.5	3.3	5.3
Morphine		4.5	4.0	4.5	3.3	5.3
Ibuprofen		4.5	4.0	4.5	3.3	5.3
Amoxicillin		13.4	9.6	12.4	8.8	5.3
Ampicillin		13.4	9.6	12.4	8.8	5.3
Benzylpenicillin		13.4	9.6	12.4	8.8	5.3
Gentamicin		13.4	9.6	12.4	8.8	5.3
Ceftriaxone		2.7	3.9	12.0	17.7	5.3
Cefotaxime		2.7	3.9	12.0	17.7	5.3
Procaine benzylpenicillin		0.2	0.5	0.1	0.3	5.3
**Total**		**100%**	**100%**	**100%**	**100%**	**100%**

A = base case scenario, using GHE code 370 as a proxy for infectious diseases. B = GHE code 370, and the burden of a disease is divided over all medicines for treating that specific disease. C = GHE code 20 (original methodology). D = GHE code 20, and the burden of a disease is divided over all medicines for treating that specific disease. E = no DB weighting (e.g. equal weights for all medicines)

Scenarios A-K were repeated for Dataset 1 and Dataset 2. The mean and range of the facility scores and the SDG 3.b.3. indicator scores were calculated for all scenarios. To increase our understanding of how the affordability dimension responds to changes in the NPL and NUNT, accessibility of individual medicines was compared across scenarios A and G to K. Expected results of scenarios on affordability and facility scores are provided in Table 1.

To determine the smallest number of medicines that must be surveyed to obtain a stable outcome for the indicator (step 1 in Fig. 2), scenario A was applied to a continuously smaller basket of medicines. For this analysis, a random medicine was removed from the basket at each repetition and the mean, SD and range of facility scores and the SDG 3.b.3 score were calculated. This analysis was performed for Dataset 1 only.

## Results

General characteristics of Dataset 1 and Dataset 2 can be found in Annex 3. For Dataset 1, the mean facility score of the base case scenario was 35.5%, with a SDG 3.b.3. score of 0%. For Dataset 2, both the mean facility score (76.3%) and SDG 3.b.3 score (40%) were considerably higher. The higher scores in Dataset 2 were the result of an increased number of medicines that were available in this dataset due to the purposeful sampling strategy as described earlier.

**Fig. 3:**
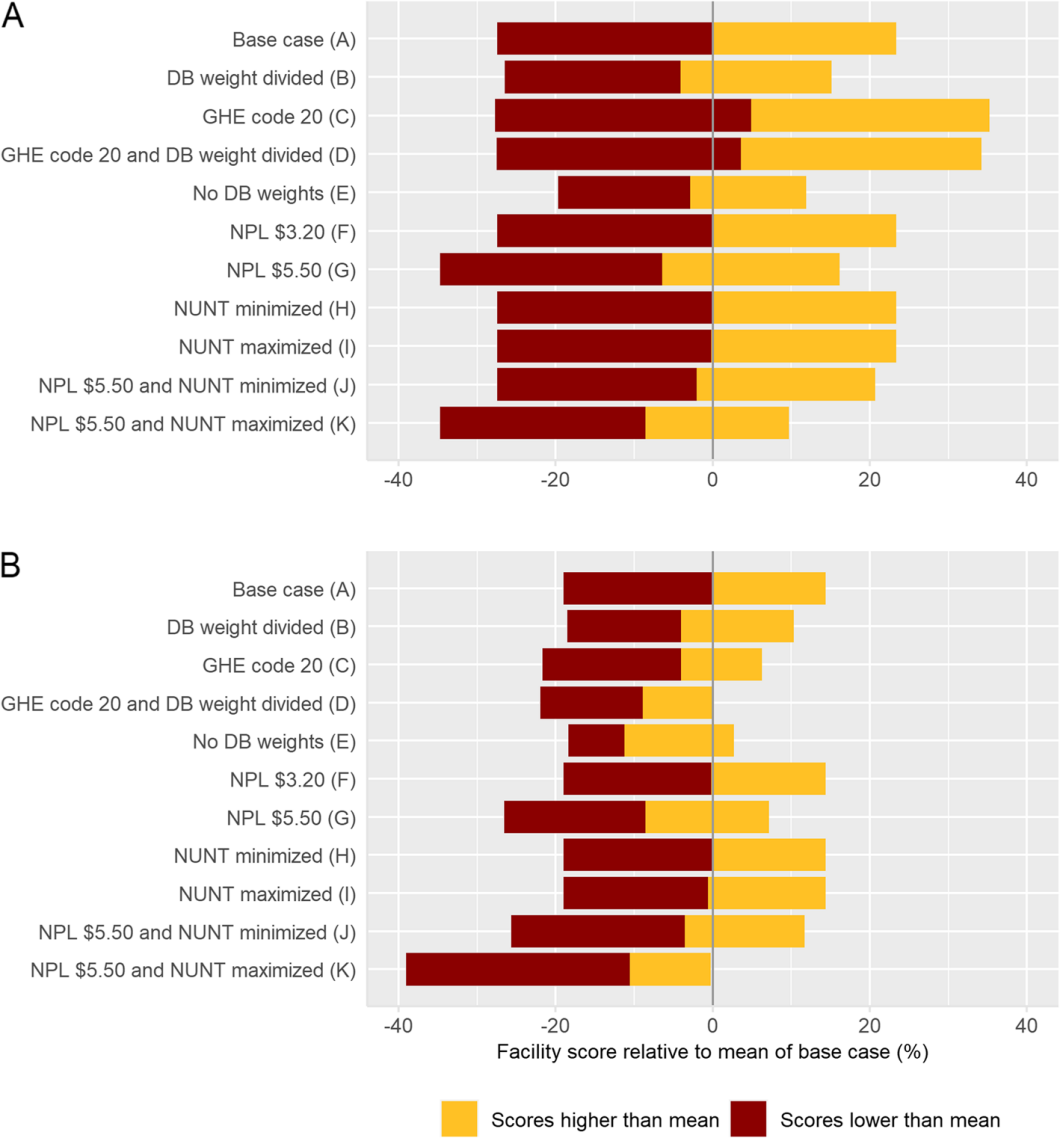
Mean, minimum and maximum facility scores of scenarios A-K. **A** = Dataset 1, **B** = Dataset 2. DB = Disease Burden; GHE = Global Health Estimates; NPL = National Poverty Line; NUNT = Number of Units Needed for Treatment

Figure 3a (Dataset 1) and b (Dataset 2) show the mean and range of the facility scores across scenarios B-K relative to the base case scenarios (A) (see also Annex 4). The ranges of facility scores in Dataset 1 were somewhat larger than those for Dataset 2, again the result of the sampling strategy.

### Weighting for burden of disease

The different weighting approaches resulted in a 9% and 11% difference in mean facility scores between scenarios for Dataset 1 and Dataset 2, respectively. These variations also had considerable effects on the minimum and especially the maximum scores observed, with changes of more than 10% in facility scores for individual facilities (see scenarios B-E in Fig. 3).

### The national poverty line

**Fig. 4:**
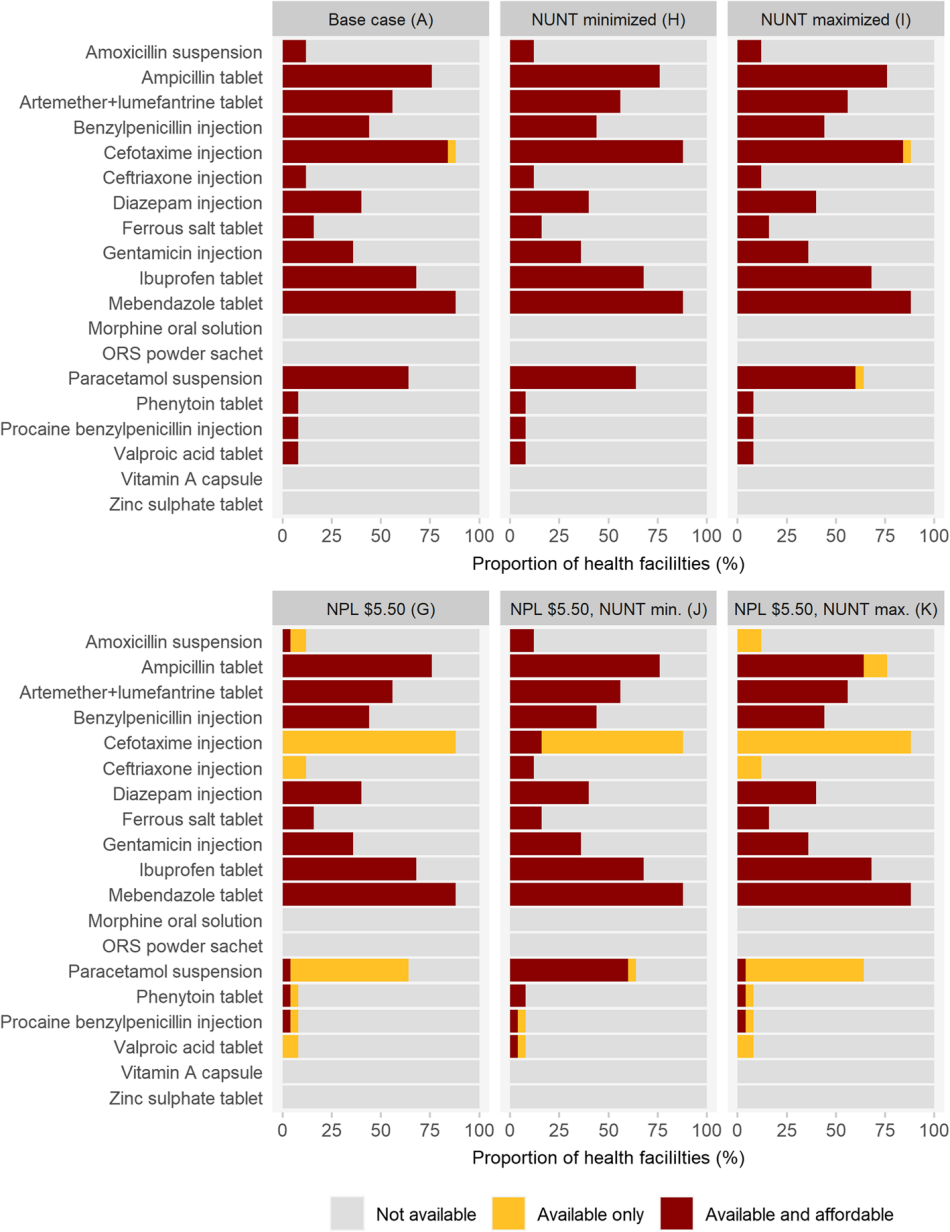
Availability and affordability of individual medicines for scenarios A and H–K for Dataset 1. NPL = National Poverty Line; NUNT = Number of Units Needed for Treatment; ORS = Oral Rehydration Salts

Increasing the NPL from $1.90 to $3.20 led to almost equal results, but a further increase to $5.50 induced an expected decline of 6.5% and 8.6% in mean facility scores for Dataset 1 and Dataset 2, respectively. Despite the only $0.54 remaining difference between NPL and LPGW wage in the latter case, the majority of medicines that were available also remained affordable (see Fig. 4).

### The number of units needed for treatment

The mean facility scores remained stable within Dataset 1 while the NUNT was varied in scenarios H and I (+ 0.1% and -0.2%), whereas differences of + 4.4% and -2.1% were observed between scenarios G and J or K at the more critical NPL of $5.50. Results were comparable for Dataset 2 (+ 0.0% (H vs. A), -0.6% (I vs. A), + 5.0 (J vs. G), -2.0% (K vs. G)). When examining the effects at the individual medicine level, the effects of changes in the NUNT can be seen in more detail (Fig. 4, Annex 5). At the poverty line of $1.90, virtually all medicines that were available were also affordable, whichever NUNT used. At the poverty line of $5.50, as expected more medicines were unaffordable, but changes to the NUNT still had limited impact. The most considerable changes were in the (un)affordability of ceftriaxone injections and paracetamol suspensions, which were also associated with a wide range in NUNT values (Annex 2).

### Size of the medicine basket

**Fig. 5:**
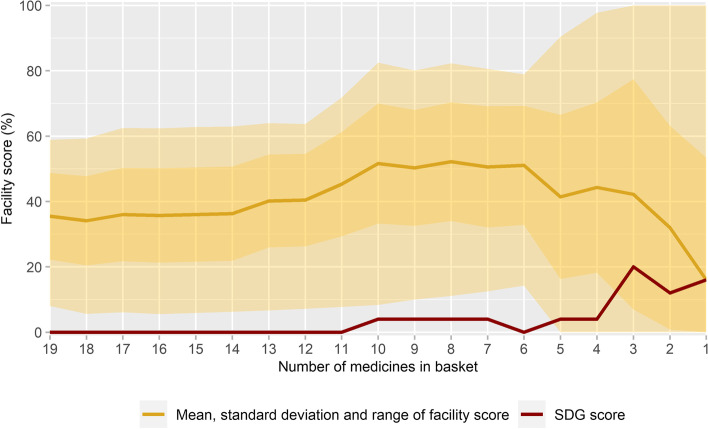
Facility scores and SDG 3.b.3 scores of an in size reducing basket. SDG = Sustainable Development Goal

Figure 5 shows the mean, SD and range of facility scores and the SDG 3.b.3 scores of a continuously reducing basket size (see also Annex 6). With a decreasing number of medicines in the basket, scores became increasingly more unstable. Less than 5% change in mean facility score was observed for baskets with at least 12 medicines. For baskets smaller than 12 medicines, mean facility scores increased more rapidly before dropping greatly and the range of scores widened further. This generated mostly moderate changes in the SDG 3.b.3 scores.

## Discussion

An adapted SDG indicator 3.b.3 methodology was developed to enable measuring of access to child medicines, but proof of its robustness had not yet been provided. With this study we aimed to provide this evidence through sensitivity analyses. These analyses have confirmed that the NUNT behaves as predicted, causing minimal to more modest variation in mean facility scores when a more critical value of the poverty line (i.e. NPL of $5.50) was used. Analyses have also demonstrated that stable results are obtained for medicine baskets of at least 12 child-appropriate medicines. Conversely, different proportional weights based on DB and higher NPL values were associated with considerable variation in facility scores.

Although there is no agreement on what degree of variation in facility scores should be considered relevant, we consider a difference in mean facility scores of less than 5% of limited influence. With that in mind, we consider the NUNT to be a reliable substitute for DDDs in this methodology. The comparable results in Dataset 1 versus Dataset 2 provide further evidence to the robustness of this parameter. Additionally, these results indicate that a single NUNT value can sufficiently represent use of a medicine in an entire age group of one month to five years. This is a crucial finding, because this indicator was designed to be inclusive of those not covered by the original indicator.

Repeated analyses on a continuously smaller number of medicines in the basket have demonstrated that there was limited variability in all outcomes when a basket of between 12 and 19 child medicines was used. Baskets smaller than 12 medicines led to increasingly diverging outcomes. Although the impact on the SDG 3.b.3 scores was still limited, larger fluctuations in SDG scores may be expected when the mean facility score (now 35.5%) is closer to the critical WHO threshold of 80% used as a reference in this methodology. The limited number of medicines in the basket could then result in more health facilities being falsely classified as (not) providing available and affordable medicines, since individual facility scores are more likely to end up just under or above the 80% threshold. We thus recommend that at least 12 medicines are used to determine access to medicines for children, although an even larger number of medicines will provide a more comprehensive picture.

In Scenario C – the weighting approach as used in the original methodology – the use of antibacterial medicines is exaggerated through the use of GHE code 20 in calculating their proportional weight. GHE code 20 is an overarching code used to represent all infectious and parasitic diseases, of which many are not treated with antibacterial medicines. With individual weights of 12.4% or 12.0%, this resulted in antibacterial medicines together accounting for 73.6% of the weighted access scores in this scenario. Medicines for pain and palliative care represented another 13.5%. If these nine medicines were available and affordable in a facility, it would be considered to provide accessible medicines, regardless of the status of the other ten medicines. In contrast, one can almost certainly not score well on this indicator if one does not meet the standards for these nine medicines. These analyses thus reveal that the scoring system that is currently part of the original SDG 3.b.3 indicator methodology is highly disproportionate towards antibacterial medicines and overstates their importance.

This study included several alternative weighting approaches that were designed to minimize this disproportionality (scenarios A-D). The different approaches led to substantial differences in weights assigned between medicines and radical shifts between scenarios for individual medicines. Each tested alternative thus also seemed to lead to disproportionate weights (i.e. no longer reflecting the actual DB). Therefore, we propose that the weighting for DB is taken out of the methodology. Instead, all medicines should be given an equal weight in the calculations (i.e. scenario E).

Besides disproportionality, there are other arguments to support this recommendation. Primarily, all medicines that are part of the core sets are essential and should thus always be available and affordable. Additionally, the weighting procedure was designed to capture the demand for medicines, yet it fails to include the volume of medicines needed to meet this demand. In this methodology, availability is a binary variable and not a continuous measure. Without quantitative availability data, demand appears to be a rather empty element. A strong argument advocating in favor of weighting for DB is that the core set includes medicines for diseases such as malaria, tuberculosis and HIV/AIDS. The DB for these diseases may be negligible in some countries. However, the medicine basket is not fixed and already allows for some flexibility. If a disease is not prevalent in a country, a country may decide not to survey these medicines. Finally, the methodology was designed for countries to apply independently, and its ease of use is thus an important factor. Removing this step from the equation will simplify its use and interpretation of the results, which we have experienced to be much needed. Of note, although the indicator currently provides no opportunities for adding medicines to the basket that are of local importance when reporting to the UN – to maintain global comparability – additional medicines may be added when this methodology is used to inform policy-making at a national or regional level.

Our results confirm that a higher value of the NPL – or in other words a smaller difference between the NPL and daily LPGW wage – led to reduced facility scores. Although international reference poverty lines were used in the present study as a proxy for the NPL, it does indicate a potential problem with the expression of affordability as a function of these parameters. We experienced first-hand that it is difficult to obtain NPL values for all countries. Values that were successfully identified were often not from the same year as the survey data, requiring additional corrections. This may be acceptable when the NPL is only a few years older, use of increasingly outdated NPL values risks severely skewed results. These problems may, however, be irrelevant to governments that have access to country data not publicly available. Notably, we also encountered an NPL that was higher than the LPGW wage (e.g. Kyrgyzstan), which would make all medicines unaffordable unless provided for free. Although this could be a testimony to reality, the use of the NPL in this indicator introduces additional uncertainties to those that already exist regarding the LPGW wage [15]. Another, more fundamental concern about the definition of affordability as used in this indicator is that it fails to consider that children do not have their own income and depend upon a caregiver for buying medicines. Methods that have been used previously to express affordability (e.g. number of days wages of the lowest-paid government worker that is needed to purchase a medicine) present the same challenge in children. The validity of the present and other methods of expressing affordability in reporting on access for children should be subject of future research [16].

Our findings show that the proposed child-specific indicator should be considered as a standard addition to the original 3.b.3 indicator in the Global Indicator Framework for tracking progress in the SDGs [5]. The issues encountered in calculating the adapted indicator are also strong predictors for similar problems in the original SDG 3.b.3 methodology as both rely on the same framework. With that, the proposed dropping of the weighting step should also be considered for the original indicator at the planned review round of the indicator framework in 2025 [17]. Until then, the same approach for weighting for DB should be used across countries and years to at least ensure comparability of results.

An important strength of this study is the pooling of data from historical datasets collected in different countries and years to obtain data on a range of medicines. This enabled us to perform a variety of sensitivity analyses on distinct aspects of the methodology, some of which would not have been possible otherwise. Although the datasets used were dated, this did not pose a limitation as it did not hinder us in our primary aim of determining the robustness of this methodology. A second dataset made it possible not only to confirm the results on different data, but also to gain additional insight into the effects of the affordability dimension. This is highly relevant, as the affordability of a medicine depends upon a large number of input variables. Although a valuable strength of the study, the pooling of data is also associated with several limitations. First, we were restricted to the use of estimates for some of the major input variables such as the LPGW wage and the NPL. Additionally, the pooling process of data from different years and countries required several correction and extraction steps that may have compromised the representability of the data. Nonetheless, the availability and affordability of medicines as observed in the present study are in line with the results of the proof-of-concept study and a recent systematic review [9, 18]. Finally, no strong conclusions about the type of medicines that are (un)affordable can be drawn as this exercise was strictly hypothetical.

## Conclusions

Including a child appropriate SDG indicator 3.b.3 in the official Global Indicator Framework is instrumental in improving access to medicines for this often neglected group. This study has confirmed that using the NUNT to express affordability for children instead of DDDs provides reliable outcomes, corroborating that the elements that were changed to make the indicator appropriate for children are robust, whilst some of the underlying principles of indicator 3.b.3 are problematic. Given its disproportionate effects, the dropping of DB from the equation should strongly be considered at the 2025 planned review of the indicator framework. Furthermore, these analyses have reinforced the need for the development of methods to measure affordability that could substitute the current calculations based on an NPL and the LPGW wage given their limitations.

## Supplementary Information

**Additional file 1: Annex 1.** Detailed description of the adapted SDG indicator 3.b.3 for children. **Table S1.** Core set of essential medicines for children 1-59 months. **Annex 2.** Number of units needed for treatment (NUNT) for children 1-59 months. **Annex 3.** General characteristics of datasets 1 and 2. **Annex 4.** Results of scenarios A-K. **Annex 5.** Availability and affordability of individual medicines for scenarios A and H to K for dataset 2. **Annex 6.** Results of analysis with reducing basket size.

## Data Availability

The individual facility data that support the findings of this study are available from Health Action International but restrictions apply to the availability of these data, which were used under license for the current study, and so are not publicly available. Aggregated data per medicine and country can be obtained from the Health Action International website. Individual facility data are available from the authors upon reasonable request and with permission of Health Action International. Author HAvdH (h.a.vandenham@uu.nl) may be contacted for such requests.

## Abbreviations

CPI: Consumer Price Index
DB: Disease Burden
DDD: Defined Daily Dosage
GHE: Global Heath Estimate
HAI: Health Action International
LPGW: Lowest-Paid unskilled Government Worker
NPL: National Poverty Line
NUNT: Number of Units Needed for Treatment
PPP: Purchasing Power Parity
SDG: Sustainable Development Goal
UN: United Nations
WHO: World Health Organization
