# Supplementary material for: SDG indicator 3.b.3 – an analysis of its robustness and challenges for measuring access to medicines for children
Source: BMC Health Serv Res. 2023 Jun 3;23:574. doi: 10.1186/s12913-023-09554-w (PMC10239173; doi:10.1186/s12913-023-09554-w)
Supplement: Supplementary file 1 — Additional file 1: Annex 1. Detailed description of the adapted SDG indicator 3.b.3 for children. Table S1. Core set of essential medicines for children 1-59 months. Annex 2. Number of units needed for treatment (NUNT) for children 1-59 months. Annex 3. General characteristics of datasets 1 and 2. Annex 4. Results of scenarios A-K. Annex 5. Availability and affordability of individual medicines for scenarios A and H to K for dataset 2. Annex 6. Results of analysis with reducing basket size. [file 12913_2023_9554_MOESM1_ESM.docx]

Annex 1

Detailed description of the adapted SDG indicator 3.b.3 for children

A core set of essential, age-appropriate medicines was established. Basis for establishing this core set was the selection of ten priority childhood diseases based on the global burden of disease. Medicines of first-choice, used in primary health care, were identified through international treatment guidelines. This primary selection of medicines was validated through expert consultation, resulting in a core set of 22 age-appropriate medicines (Table S1). This basket represents medicines for acute and chronic, communicable and non-communicable diseases in the primary health care setting.

Availability is determined for each medicine in the basket. Availability is a binary variable and is determined by the presence of the medicine on the day that the data collector visited the facility.

Affordability is determined based on the price of treatment per day and the ability to pay, which is determined by the National Poverty Line (NPL) and the Lowest-Paid unskilled Government Worker (LPGW) wage.

The price of treatment per day is determined by the unit price and the number of units needed for a course or month treatment (NUNT). Of note, the price of a medicine is only reported when the medicine was available.

$$Price per treatment (day)=\frac{unit price (day)*NUNT (month)}{365/12}$$

A medicine is considered affordable when no extra daily wages (EDW) are required for the LPGW to purchase a daily dose treatment of this medicine after fulfilling basic needs (represented by the NPL). The closer the NPL value and LPGW wage are to each other, the cheaper a medicine has to be to still be affordable.

$$Extra daily wages \left( EDW \right)= \frac{NPL+price per treatment (day)}{daily wage of LPGW}$$

The EDW is transformed into a binary variable.

$$\left\{ \begin{aligned} if EDW \leq1, affordability = 1, \\ otherwise, affordability = 0 \end{aligned} \right.$$

Of note, this indicator is used to represent the patient (or parent) perspective. If a medicines is provided for free by a government or donor program, it should be given a price of ‘0’ in the calculations. It should then automatically be regarded as affordable.

Medicines are only considered accessible when they are both available and affordable in a facility. Accessible medicines are given a relative weight (between 0-100%) according to the regional burden of disease to capture demand. Weights are determined through Disability Adjusted Life Years (DALYs) as can be found in the WHO Global Health Estimates (GHE).

Relative weights applied to each individual medicine can be calculated through the following steps:

1. Each medicine in the basket is assigned a GHE code for one or several disease(s) that are treated/cured/controlled by that medicine (table S1).
2. Each medicine is assigned the corresponding number of disability-adjusted life years (DALYs). If a medicine is used to treat multiple diseases, the DALYs for these diseases are summed.*
3. The total number of DALYs for the basket is calculated.
4. The proportional weight per medicine is calculated as the number of DALYs linked to each individual medicine divided by the total number of DALYs.
5. Medicines used in the treatment of pain and palliative care cannot be linked to a GHE code. Weights for these medicines are thus calculated by dividing 1 by the number of medicines in the basket.
6. The proportional total is calculated by summing the proportional weights from steps 4 and 5.
7. Final weights are calculated by dividing the proportional weights from step 4 and 5 by the proportional total.

*If a diseases is associated with multiple medicines, the burden for this specific is thus counted multiple times.

Facility scores are generated (0-100%), representing weighted access scores for individual health facilities. A facility provides access to medicines if 80% of the medicines are available and affordable, transforming it to a binary variable.

$$\left\{ \begin{aligned} if facility score \geq80\%, accessibility = 1, \\ otherwise, accessibility = 0 \end{aligned} \right.$$

The adapted SDG indicator 3.b.3 score can be calculated as the number of health facilities with an available and affordable core set of child medicines divided by the total number of health facilities surveyed.

$${SDG}_{3.b.3.}\text{ }\text{= }\frac{Facilities with available and affordable basket of medicines (n)}{Surveyed Facilities (n)}$$

**Table S1** Core set of essential medicines for children 1-59 months

| **Affiliated disease (GHE code)** | **Medicine name** | **Acceptable formulations** |
| --- | --- | --- |
| Diarrhoeal diseases (110) | Oral rehydration salts | *Powder sachet 200 ml, 500 ml or 1L* |
|  | Zinc sulphate | *Cap/tab 20 mg* |
| Epilepsy (970) | Carbamazapine | *Cap/tab 100 mg; oral liquid 100 mg/5 ml* |
|  | **OR** Phenobarbital | *Cap/tab 30 mg or 100 mg; injection 100 mg/ml or 200 mg/ml; oral liquid 15 mg/5 ml* |
|  | **OR** Phenytoin | *Cap/tab 25 mg, 50 mg or 100 mg; injection 50 mg/ml; oral liquid 25 or 30 mg/5 ml* |
|  | **OR** Lamotrigine | *Cap/tab 25 mg, 50 mg or 100 mg* |
|  | Valproic acid | *Cap/tab 100 mg, 150 mg, 200 mg or 500 mg; oral liquid 200 mg/5 ml* |
|  | Diazepam | *Rectal solution 5 mg/ml; injection 5 mg/ml* |
|  | **OR** Lorazepam | *Parenteral solution 2 mg/ml or 4 mg/ml* |
|  | **OR** Midazolam | *Oromucosal solution 5 mg/ml or 10 mg/ml; ampoule 10 mg/ml* |
| HIV/AIDS (100) | Abacavir + lamivudine + dolutegravir | *Cap/tab 120/60 mg (abacavir/lamivudine)* ***AND*** *cap/tab 10 mg (dolutegravir)* |
|  | **OR** Abacavir + lamivudine + lopinavir/ritonavir | *Cap/tab 120/60 mg (abacavir/lamivudine)* ***AND*** *cap/tab 40/10 mg or 100/25 mg (lopinavir/ritonavir)* |
| Iron-deficiency anemia (580) | Ferrous salt | *Cap/tab 60 mg or 200 mg; oral liquid 25 mg/ml* |
|  | Albendazole | *Cap/tab 200 mg or 400 mg* |
|  | **OR** Mebendazole | *Cap/tab 100 mg* |
| Malaria (220) | Artemether + lumefantrine | *Cap/tab 20/120 mg* |
|  | **OR** Artesunate + amodiaquine | *Cap/tab 25/67.5 mg or 50/135 mg* |
|  | **OR** Artesunate + mefloquine | *Cap/tab 25/55 mg* |
|  | **OR** Dihydroartemisinin + piperaquine | *Cap/tab 20/160 mg or 20/320 mg* |
|  | **OR** Artesunate + Sulfadoxine-pyrimethamine | *Cap/tab 50/500/25 mg or cap/tab 50 mg (artesunate)* ***AND*** *cap/tab 500/25 mg (sulfadoxine-pyrimethamine)* |
|  | **OR** Chloroquine | *Cap/tab 100 mg; oral liquid 50 mg/5 ml* |
|  | Artesunate | *Cap/tab 50 mg; suppository 50 mg* |
| Measles (150)  Vitamin A deficiency (570) | Retinol | *Cap/tab 25,000 IU, 100,000 IU or 200,000 IU* |
| Pain and palliative care  (weight = 1/T) | Paracetamol | *Cap/tab 100 mg; suppository 100 mg; suspension 120 or 125 mg/5 ml* |
|  | Morphine | *Cap/tab (slow release) 10 mg; injection 10 mg/ampoule; oral liquid 10 mg/5 ml* |
|  | Ibuprofen | *Cap/tab 200 mg; oral liquid 200 mg/5 ml* |
| Tuberculosis (30) | Ethambutol + isoniazid + pyrazinamide + rifampicin | *Cap/tab 100 mg or 400 mg or oral liquid 25 mg/ml (ethambutol)* ***AND*** *cap/tab 50/150/75 mg (isoniazid + pyrazinamide + rifampicin)* |
| Lower respiratory infections (390)  Other infectious diseases (370) | Amoxicillin | *Cap/tab 250 mg or 500 mg; powder for injection 250 mg/vial, 500 mg/vial or 1 g/vial; suspension 125 mg/5 ml or 250 mg/5 ml* |
|  | **OR** Amoxicillin + clavulanic acid | *Cap/tab 100/125 mg, 250/125 mg or 500/125 mg; powder for injection 500/100 mg/vial; oral liquid 125/53.25 mg/5 ml or 250/62.5 mg/5 ml* |
|  | Ampicillin | *Cap/tab 250 mg or 500 mg; injection 500 mg/vial or 1 g/vial* |
|  | Benzylpenicillin | *Injection 1 MIU/vial* |
|  | Gentamicin | *Injection 10 mg/ml or 40 mg/ml* |
| Other infectious diseases (370)  Meningitis (170) | Ceftriaxone | *Injection 250 mg/vial, 500 mg/vial or 1 g/vial* |
|  | Cefotaxime | *Injection 1 g/vial* |
| Syphilis (50) | Procaine benzylpenicillin | *Injection 1 MIU/vial* |

Cap/tab = capsule/tablet

Annex 2

**Table S2** number of units needed for treatment (NUNT) for children 1-59 months

| **Medicine name** | **Formulation** | **NUNT** | **Minimum** | **Maximum** |
| --- | --- | --- | --- | --- |
| Amoxicillin | Suspension 250 mg/5 ml | 90 | 30 | 100 |
| Ampicillin | Cap/tab 500 mg | 20 | 10 | 40 |
| Artemether/lumefantrine | Cap/tab 20/120 mg | 6 | 3 | 12 |
| Artesunate/Sulfadoxine-pyrimethamine | Cap/tab 50/500/25 mg | 1 | 1 | 1 |
| Benzylpenicillin | Injection 1 MIU/vial | 5 | 5 | 5 |
| Cefotaxime | Injection 1 g/vial | 18 | 7 | 30 |
| Ceftriaxone | Injection 250 mg/vial | 28 | 7 | 40 |
| Diazepam | Injection 5 mg/ml | 1 | 1 | 1 |
| Ferrous salt | Cap/tab 200 mg | 14 | 7 | 14 |
| Gentamicin | Injection 40 mg/ml | 10 | 4 | 15 |
| Ibuprofen | Cap/tab 200 mg | 90 | 45 | 120 |
| Mebendazole | Cap/tab 100 mg | 6 | 6 | 6 |
| Morphine | Oral liquid 10 mg/5 ml | 300 | 60 | 720 |
| Oral rehydration salts | Powder sachet 500 ml | 2 | 1 | 6 |
| Paracetamol | Suspension 120 mg/5 ml | 900 | 240 | 1800 |
| Phenytoin | Cap/tab 50 mg | 60 | 30 | 120 |
| Procaine benzylpenicillin | Injection 1 MIU/vial | 10 | 10 | 10 |
| Valproic acid | Cap/tab 150 mg | 60 | 30 | 60 |
| Vitamin A | Cap/tab 100,000 IU | 2 | 1 | 6 |
|  | Cap/tab 200,000 IU | 2 | 1 | 3 |
| Zinc sulphate | Cap/tab 20 mg | 14 | 5 | 14 |

Cap/tab = Capsule/tablet; IU = international units.
The standard NUNT is based on a 30 months old child of 11 kg, the minimum and maximum NUNT values loosely correspond to a 1 month old child of 4 kg and a 5 year old of 18 kg respectively.

Annex 3

**Table S3** General characteristics of datasets 1 and 2.

| **Dataset 1** | | | **Dataset 2** | | |
| --- | --- | --- | --- | --- | --- |
| **Medicine** | **Formulation** | **Original dataset** | **Medicine** | **Formulation** | **Original dataset** |
| Amoxicillin | Suspension 50 mg/ml | Haiti (2011) | Amoxicillin | Suspension 50 mg/ml | Burundi (2013) |
| Ampicillin | Cap/tab 500 mg | Mongolia (2004) | Ampicillin | Cap/tab 500 mg | Mongolia (2004) |
| Artemether/lumefantrine | Cap/tab 20/120 mg | Tanzania (2012) | Artesunate/sulfadoxine/  pyrimethamine | Cap/tab 50/500/25 mg | Sudan (2012) |
| Benzylpenicillin | Injection 1 MIU/vial | China (2012) | Benzylpenicillin | Injection 1 MIU/vial | China (2012) |
| Cefotaxime | Injection 1 g/vial | Bolivia (2008) | Cefotaxime | Injection 1 g/vial | Bolivia (2008) |
| Ceftriaxone | Injection 250 mg/vial | China (2012) | Ceftriaxone | Injection 250 mg/vial | Mongolia (2004) |
| Diazepam | Injection 5 mg/ml | Tanzania (2012) | Diazepam | Injection 5 mg/ml | Tanzania (2012) |
| Ferrous salt | Cap/tab 200 mg | Tanzania (2012) | Ferrous salt | Cap/tab 200 mg | Tanzania (2012) |
| Gentamicin | Injection 40 mg/ml | Burundi (2013) | Gentamicin | Injection 40 mg/ml | Kyrgyzstan (2015) |
| Ibuprofen | Cap/tab 200 mg | Kyrgyzstan (2010) | Ibuprofen | Cap/tab 200 mg | Haiti (2011) |
| Mebendazole | Cap/tab 100 mg | Bolivia (2008) | Mebendazole | Cap/tab 100 mg | Burundi (2013) |
| Morphine | Oral solution 2 mg/ml | Haiti (2011) | Morphine | Oral solution 2 mg/ml | Haiti (2011) |
| Oral rehydration salts | Powder sachet 500 ml | Haiti (2011) | Oral rehydration salts | Powder sachet 500 ml | China (2012) |
| Paracetamol | Suspension 24 mg/ml | Haiti (2011) | Paracetamol | Suspension 24 mg/ml | Sudan (2013) |
| Phenytoin | Cap/tab 50 mg | Haiti (2011) | Phenytoin | Cap/tab 50 mg | China (2012) |
| Procaine benzylpenicillin | Injection 1 MIU/vial | Haiti (2011) | Procaine benzylpenicillin | Injection 1 MIU/vial | Kyrgyzstan (2015) |
| Valproic acid | Cap/tab 150 mg | Kyrgyzstan (2010) | Valproic acid | Cap/tab 150 mg | Kyrgyzstan (2010) |
| Vitamin A | Cap/tab 200,000 IU | Burundi (2013) | Vitamin A | Cap/tab 100,000 IU | Haiti (2011) |
| Zinc sulphate | Cap/tab 20 mg | Burundi (2013) | Zinc sulphate | Cap/tab 20 mg | Haiti (2011) |

Annex 4

**Table S4** Results of scenarios A-K. All results are in percentages (%).

|  | **Results dataset 1** | | | **Results dataset 2** | | |
| --- | --- | --- | --- | --- | --- | --- |
| **Scenario** | **Mean FS** | **Minimum FS** | **Maximum FS** | **Mean FS** | **Minimum FS** | **Maximum FS** |
| A | 35.5 | 8.0 | 58.8 | 76.3 | 57.2 | 90.6 |
| B | 31.4 | 9.0 | 50.6 | 72.2 | 57.7 | 86.6 |
| C | 40.4 | 7.8 | 70.7 | 72.2 | 54.9 | 82.5 |
| D | 39.1 | 7.9 | 69.6 | 67.3 | 54.3 | 76.4 |
| E | 32.6 | 15.8 | 47.4 | 65.1 | 57.9 | 78.9 |
| F | 35.5 | 8.0 | 58.8 | 76.0 | 57.2 | 90.6 |
| G | 29.0 | 0.8 | 51.6 | 67.7 | 49.7 | 83.4 |
| H | 35.6 | 8.0 | 58.8 | 76.3 | 57.2 | 90.6 |
| I | 35.3 | 8.0 | 58.8 | 75.7 | 57.4 | 90.6 |
| J | 33.4 | 8.0 | 56.1 | 72.7 | 50.6 | 87.9 |
| K | 26.9 | 0.8 | 45.2 | 65.7 | 37.2 | 76.0 |

FS = Facility score

Annex 5


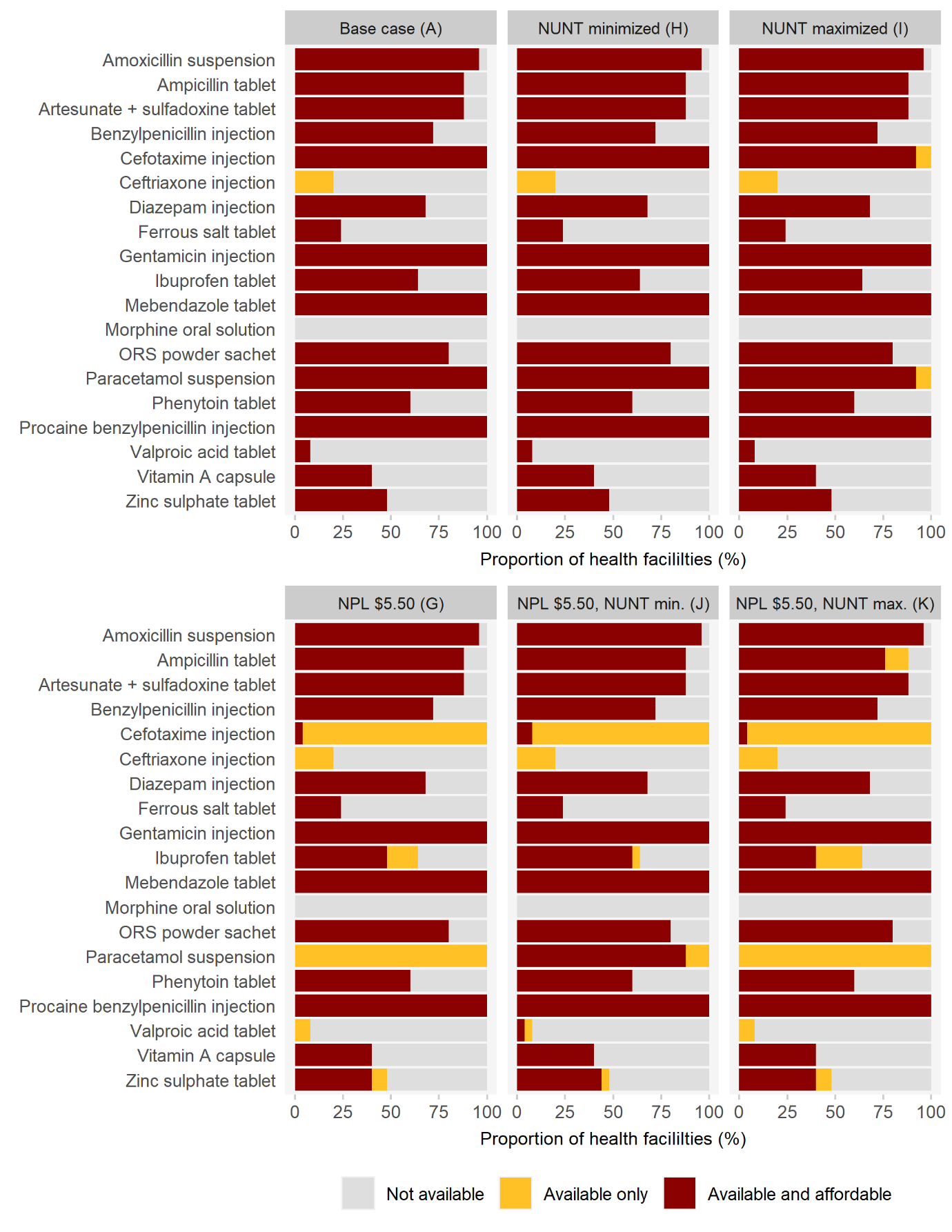


**Figure S1** Availability and affordability of individual medicines for scenarios A and H to K for dataset 2. NPL = National Poverty Line; NUNT = Number of Units Needed for Treatment; ORS = Oral Rehydration Salts.

Annex 6

**Table S5** Results of analysis with reducing basket size. All results are in percentages (%).

| **Number of medicines in basket** | **Mean** | **SD** | **Minimum** | **Maximum** | **SDG score** |
| --- | --- | --- | --- | --- | --- |
| 1 | 16.0 | 37.4 | 0.0 | 100.0 | 16.0 |
| 2 | 32.0 | 31.2 | 0.0 | 100.0 | 12.0 |
| 3 | 42.2 | 35.2 | 0.0 | 100.0 | 20.0 |
| 4 | 44.3 | 26.0 | 0.0 | 97.8 | 4.0 |
| 5 | 41.4 | 25.1 | 0.0 | 90.4 | 4.0 |
| 6 | 51.0 | 18.2 | 14.3 | 78.9 | 0.0 |
| 7 | 50.6 | 18.5 | 12.5 | 80.6 | 4.0 |
| 8 | 52.2 | 18.2 | 11.1 | 82.3 | 4.0 |
| 9 | 50.3 | 17.7 | 10.0 | 80.1 | 4.0 |
| 10 | 51.6 | 18.4 | 8.3 | 82.5 | 4.0 |
| 11 | 45.3 | 16.0 | 7.7 | 71.8 | 0.0 |
| 12 | 40.4 | 14.1 | 7.1 | 63.7 | 0.0 |
| 13 | 40.1 | 14.2 | 6.7 | 63.9 | 0.0 |
| 14 | 36.3 | 14.4 | 6.3 | 62.9 | 0.0 |
| 15 | 36.0 | 14.4 | 5.8 | 62.7 | 0.0 |
| 16 | 35.7 | 14.4 | 5.6 | 62.3 | 0.0 |
| 17 | 36.0 | 14.3 | 6.1 | 62.5 | 0.0 |
| 18 | 34.1 | 13.6 | 5.6 | 59.3 | 0.0 |
| 19 | 35.5 | 13.2 | 8.0 | 58.8 | 0.0 |

SD = standard deviation, SDG = sustainable development goal
